# Methionine Adenosyltransferase 1a (MAT1A) Enhances Cell Survival During Chemotherapy Treatment and is Associated with Drug Resistance in Bladder Cancer PDX Mice

**DOI:** 10.3390/ijms20204983

**Published:** 2019-10-09

**Authors:** Kelly A. Martin, Nicholas R. Hum, Aimy Sebastian, Wei He, Salma Siddiqui, Paramita M. Ghosh, Chong-xian Pan, Ralph de Vere White, Gabriela G. Loots

**Affiliations:** 1 Lawrence Livermore National Laboratory, Physical and Life Sciences Directorate, Livermore, CA 94550, USA; martin249@llnl.gov (K.A.M.); hum3@llnl.gov (N.R.H.); sebastian4@llnl.gov (A.S.); he4@llnl.gov (W.H.); 2 Department of Biochemistry & Molecular Biology, Georgetown University, Washington, DC 20057, USA; 3 UC Merced, School of Natural Sciences, Merced, CA 95343, USA; 4 UC Davis Comprehensive Cancer Center, Sacramento, CA 95817, USA; SALMA.SIDDIQUI@va.gov (S.S.); paghosh@ucdavis.edu (P.M.G.); cxpan@ucdavis.edu (C.-x.P.); rwdeverewhite@ucdavis.edu (R.d.V.W.)

**Keywords:** bladder cancer, drug resistance, methyltransferase, gemcitabine

## Abstract

Bladder cancer is among the top ten most common cancers, with about ~380,000 new cases and ~150,000 deaths per year worldwide. Tumor relapse following chemotherapy treatment has long been a significant challenge towards completely curing cancer. We have utilized a patient-derived bladder cancer xenograft (PDX) platform to characterize molecular mechanisms that contribute to relapse following drug treatment in advanced bladder cancer. Transcriptomic profiling of bladder cancer xenograft tumors by RNA-sequencing analysis, before and after relapse, following a 21-day cisplatin/gemcitabine drug treatment regimen identified methionine adenosyltransferase 1a (*MAT1A*) as one of the significantly upregulated genes following drug treatment. Survey of patient tumor sections confirmed elevated levels of *MAT1A* in individuals who received chemotherapy. Overexpression of *MAT1A* in 5637 bladder cancer cells increased tolerance to gemcitabine and stalled cell proliferation rates, suggesting *MAT1A* upregulation as a potential mechanism by which bladder cancer cells persist in a quiescent state to evade chemotherapy.

## 1. Introduction

Bladder cancer is the eighth most common type of cancer in the United States, with an estimated 80,000 new cases reported each year [1]. The stage of disease at the time of diagnosis is critical for determining patient outcome; the survival rate for cancers found at early stages is greater than 85%. However, once the cancer has reached stages three or four and has progressed from the bladder to the abdominal cavity or lymph nodes, the five-year survival rate drops below 50% [2]. Cancer becomes increasingly challenging to treat as it metastasizes to other tissues. Routine standard of care with first line chemotherapeutics typically result in tumor shrinkage, however, most cases relapse despite initial chemotherapeutic sensitivity and the recurring tumor is usually drug resistant [3]. For almost all cancer types, the emergence of drug resistance has long been a challenge in achieving a complete cure. This phenomenon is widespread in that almost all cancers can become drug resistant and cancer cells can become resistant to almost all types of therapeutic agents. Chemotherapy resistant cancer cells then progress into more aggressive tumors, capable of both metastasizing to distant sites and withstanding future drug treatments, ultimately contributing to a patient mortality [4].

Advanced bladder cancer is commonly treated with nonspecific chemotherapeutics, such as cisplatin and gemcitabine [3]. Cisplatin belongs to the platinum-based family of chemotherapeutics while gemcitabine is a type of DNA synthesis inhibitor. While having slightly different mechanisms of action, both rely on the rapidly dividing nature of cancer cells. Gemcitabine is an analog of deoxycytidine that once inside a cell it becomes activated via phosphorylation, to be used as a nucleoside during DNA synthesis. Inhibition of DNA synthesis is the most likely mechanism by which gemcitabine causes cell death. Gemcitabine nucleotide incorporation into the elongating DNA strand stalls the DNA polymerase. This action locks the drug onto the DNA rendering proofreading enzymes incapable of removing gemicitabine-DNA adducts. Previous research has shown that high levels of gemcitabine-DNA adducts in bladder cancer patients correlate with drug response [5]. While gemcitabine is a well-known chemotherapeutic agent, the molecular mechanisms by which cells are rendered chemoresistant post remission have yet to be fully elucidated. Additionally, dormancy-type mechanisms by which cells evade drug treatment can be applicable to other first line nonspecific therapies that do not depend on cancer cell proliferation to render an effect.

Methionine adenosyltransferase 1a (*MAT1A*) is an enzyme that helps regulate an important biological molecule, S-adenosylmethionine (SAM) that plays a key role in the methylation cycle [6]. The importance of S-adenosylmethionine has been a topic of increased interest as it relates to metabolism and dysregulation of cancer cells [7,8], however, direct links between this protein and enhanced survival during chemotherapy treatment have yet to be established. Here, we found that upregulation of *MAT1A* correlates with increased resistance to gemcitabine treatment. Methyltransferases of various types have been previously implicated in cancer progression, however for the first time, we showed that *MAT1A* elevated levels confer a significant survival advantage in bladder cancer cells during drug exposure. Here, we utilized RNA sequencing (RNA-seq) to identify transcripts differentially expressed in drug relapsed patient-derived bladder cancer xenograft (PDX) tumors of bladder cancer when compared to untreated tumors. We determined that transient upregulation of *MAT1A* in response to drug exposure allowed cells to adopt a less proliferative state. This work has provided novel insights into the temporal nature of gene regulation in response to nonspecific chemotherapy exposure. Furthermore, *MAT1A* may have clinical applications as a biomarker of a drug resistant cell subpopulation.

## 2. Results

### 2.1. Significant Changes in the Transcriptome of Bladder Cancer Patient-Derived Xenografts are Associated with Drug Relapse Following Treatment.

**Figure 1:**
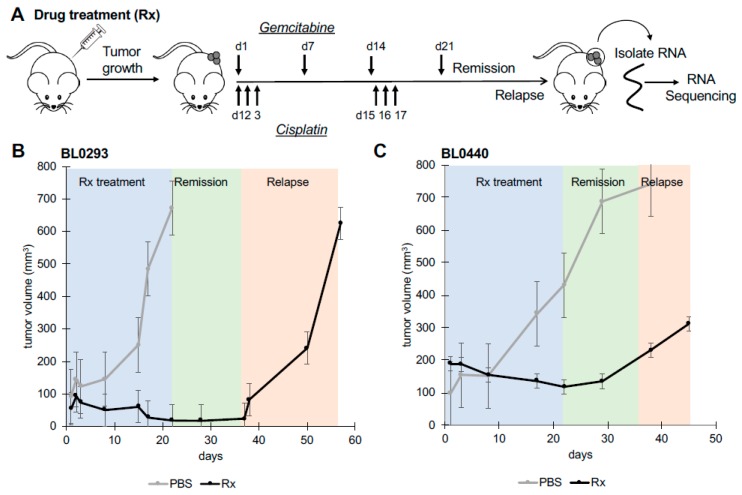
Patient-derived bladder cancer xenograft (PDX) tumors BL0293 and BL0440 relapse following combination drug therapy. (**A**) Drug dosing was initiated when tumors reached ~100–150 mm^3^ in size and occurred over 21 days with experimental mice (drug treated (Rx)) (*n* = 5) and phosphate buffered saline (PBS) control mice (*n* = 3) treated concurrently with gemcitabine (intraperitoneally) and cisplatin (intravenously). Tumor growth was measured weekly for (**B**) BL0293 and (**C**) BL0440. RNA was isolated from PBS and Rx tumors at last time point recorded.

**Figure 2:**
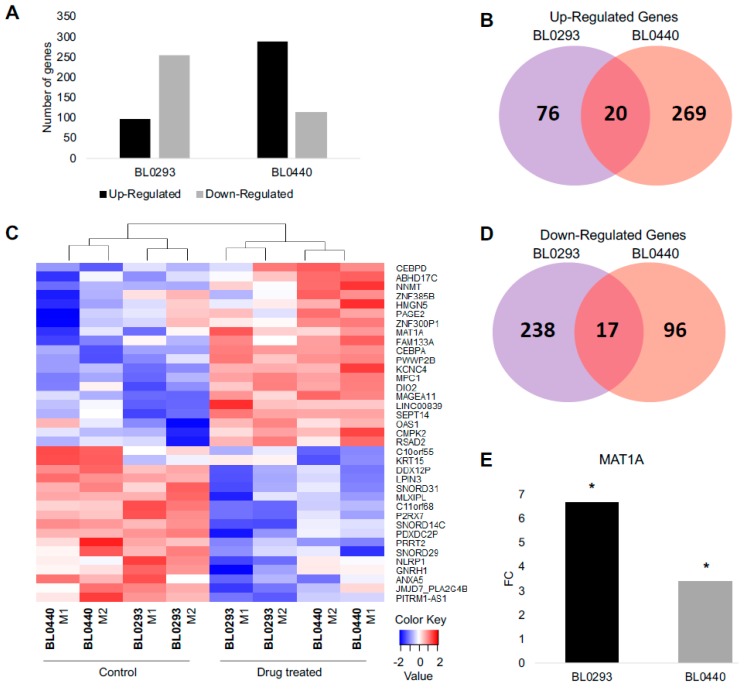
RNA sequencing (RNA-seq) profiling identifies significant transcriptional changes in drug treated bladder PDX tumors. (**A**) 351 and 402 genes were found significantly changed in BL0293 and BL0440 drug-relapsed tumors; (**B**) 20 up-regulated and (**D**) 17 down-regulated genes where shared by both drug-relapsed xenografts. (**C**) A heat map depicts variation of shared up- and down-regulated genes among BL0293 and BL0440 biological replicates. M1 and M2 denote biological replicates. (**E**) methionine adenosyltransferase 1a (*MAT1A*) gene expression values in sensitive (PBS) and drug treated (Rx) tumors from RNA sequencing data, expressed in fold change (FC), for both BL0293 and BL0440.

To identify molecular changes associated with chemotherapeutic resistance, RNA-seq was performed on PDX tumors derived from human bladder cancer cells. Tumors were initiated by delivering 3 × 10^6^ tumor cells subcutaneously into NOD *scid* gamma (NSG) mice from two patient tumor lines, BL0293 and BL0440, previously shown to be sensitive to cisplatin (CIS) and gemcitabine (GEM) combination therapy [9]. A clinically relevant regimen of CIS/GEM was administered when tumor size reached 100–150 mm^3^ and RNA was harvested and analyzed when relapsed tumors reached ~700 mm^3^ in size or when other Institutional Animal Care and Use Committee (IACUC) criteria were met (Figure 1A). Both tumors initially responded to treatment as evidenced by a decrease in tumor size and a reduction in growth rate during CIS/GEM administration; however, both lines relapsed and grew at an accelerated rate after 35 days (Figure 1B,C), similar to other human-bladder cancer PDX lines tested for sensitivity to chemotherapy [9]. The BL0293 tumors however, grew at much faster rate than the BL0440 tumors, after the remission period (Figure 1B,C). The transcriptome from these relapsed tumors was profiled and compared to phosphate buffered saline (PBS) treated tumors to identify differentially expressed genes in relapsed tumors. In total, there were 96 genes that were significantly upregulated (FDR *p* < 0.05) in BL0293 following chemotherapy and 255 genes that were significantly downregulated. To a similar extent, we identified 289 transcripts significantly upregulated and 113 genes significantly downregulated in BL0440 relapsed tumors (Figure 2A).

We found 37 genes to be significantly changed in response to drug treatment, in both models of bladder cancer drug relapse (Figure 2B–D). Among the upregulated genes, there were 20 genes that were upregulated when both PDX BL0293 and BL0440 relapsed following drug treatment, with 76 genes being upregulated only in BL0293 and 269 genes uniquely upregulated in BL0440 (Figure 2B). Additionally, there were 17 genes downregulated in both PDX models, with 238 genes downregulated in response to drug treatment in tumor BL0293 and 96 genes uniquely downregulated when tumor BL0440 relapsed (Figure 2D). Common genes found to be most differentially expressed following chemotherapeutic administration are listed in Table 1 and Table S1. *MAT1A* was one of the top significantly upregulated genes with a fold change (FC) value of 6.66 and 3.40 once BL0293 and BL0440 tumors relapsed following treatment, respectively (Figure 2E).

Seven enriched pathways were determined from the 20 genes upregulated in both BL0293 and BL0440 relapsed tumors (Table 2). Top pathway ontologies enriched included ‘Interferon alpha-beta signaling’, ‘Metabolism’, ‘Transcriptional regulation’, and ‘Methylation’. *MAT1A* was included in several top ontologies including ‘Metabolism of ingested SeMet, Sec, MeSec into H2Se’, ‘Methylation’, ‘Metabolism of amino acids and derivatives’, ‘Phase II conjugation’, and ‘Selenoamino acid metabolism’. Top pathway ontologies based on shared downregulated genes include ‘Inflammasomes’ and ‘PKA-mediated phosphorylation of key metabolic factors’.

### 2.2. MAT1A Expression Increases in Response to Chemotherapy in Bladder Cancer and Confers a Survival Advantage

**Figure 3:**
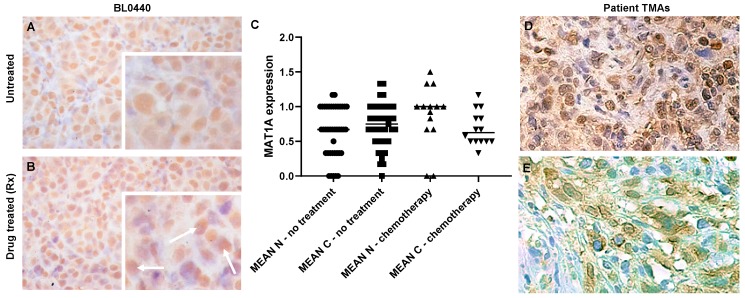
*MAT1A* protein levels increase in drug relapsed PDX and clinical patient tumors. *MAT1A* protein expression was examined by immunohistochemical (IHC) analysis in (**A**) non-drug relapsed and (**B**) drug relapsed BL0440 tumors. Images taken at 40× magnification, insets are enlargements of image shown, white arrows highlighting regions of enhanced *MAT1A* abundance in chemotherapy treated tumors. (**C**) *MAT1A* expression quantification across patient tissue microarrays (TMAs) (n, nuclear; c, cytoplasmic). (**D**,**E**) Evaluation of TMA of bladder cancer samples obtained during cystectomy. IHC analysis includes *MAT1A* protein stained with labeled streptavidin biotin (LSAB) and counterstained with hematoxylin. (**D**) Sample showing nuclear and cytoplasmic localization of *MAT1A* from male patient receiving six cycles of chemotherapy which included gemcitabine; (**E**) sample showing only cytoplasmic *MAT1A* localization from male patient receiving Bacillus Calmette-Guerin (BCG).

We identified *MAT1A* as one of the top significantly upregulated genes with a fold change value of 6.66 and 3.40 in BL0293 and BL0440 relapsed tumors, respectively (Figure 2E). This finding was validated through immunohistochemical analysis showing increased *MAT1A* protein abundance in drug treated tumors (Figure 3B) compared to untreated controls (Figure 3A) and patient bladder tumor tissue sections having received neoadjuvant chemotherapy (Figure 3D,E).

*MAT1A* is a low abundance transcript detected by RNA in a few human tissues (Figure S1A; liver, ovary, pancreas, and testes) with evidence of high protein expression by immunohistochemistry (IHC) in the human liver (Figure S1B) [10]. However, the human bladder has virtually undetectable *MAT1A* RNA and protein levels, natively (Figure S1A,C) [10,11]. As such, *MAT1A* RNA expression is not endogenous to any of the cells residing in the healthy urinary bladder, and *MAT1A* transcription initiates in bladder cancer cells after the epithelia undergo oncogenic transformation [9]. To determine whether *MAT1A* expression found in PDX models of bladder cancer relapse is also broadly observed in tumors harvested from patients that received chemotherapy, we examined *MAT1A* protein expression by IHC in bladder cancer biopsies from 55 patients (Table S2). The median age of these patients was 68 (36–85) and 81.8% were male, consistent with the reported higher incidence rates in men [12]. Eleven of 55 (20%) patients received chemotherapy, whereas 4/55 (7.27%) received Bacillus Calmette-Guerin (BCG) as neoadjuvant therapy. We observed an increase in the nuclear, but not the cytoplasmic IHC staining for *MAT1A* (Mean: 0.966 ± 0.42) compared to those whose prior treatment status was listed as “not reported” (Mean: 0.6468 ± 0.3568) or those previously treated with BCG (Mean: 0.6475 ± 0.454); this difference was not quite significant (*p* = 0.0572), likely due to insufficient statistical power from a limited sample size (Figure 3C). Figure 3D illustrates the typical IHC pattern observed for patients sample with both cytoplasmic and nuclear staining, while Figure 3E illustrates human samples with predominant cytoplasmic staining. However, our findings revealed that 100% of the patient samples with reported neoadjuvant chemotherapy had some level of *MAT1A* protein expression, suggesting that *MAT1A* protein expression correlates with chemotherapy treatment in human bladder tumors.

**Figure 4:**
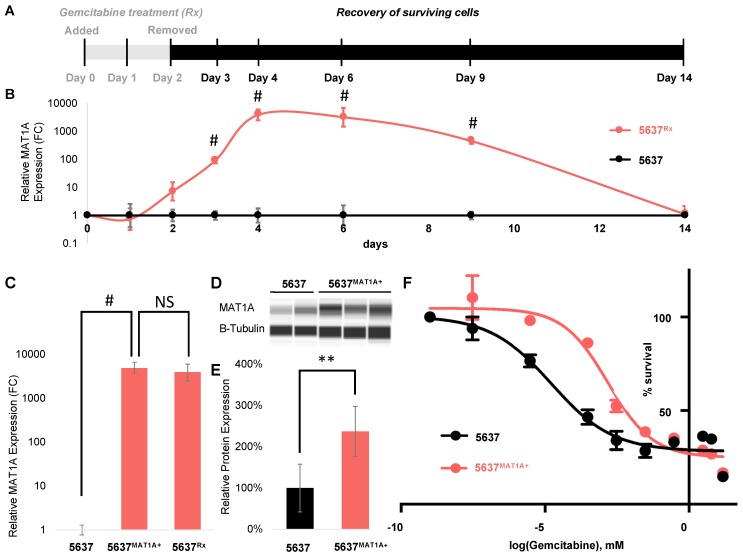
*MAT1A* is upregulated in cells that survive 48 h drug treatment and overexpression alters cell response to gemcitabine. (**A**) 5637 cells were dosed at ~80% IC50 gemcitabine concentration (0.3 µM) for 48 h in vitro. RNA was collected from remaining gemcitabine treated (5637^Rx^, *n* = 3) and untreated cells (5637, *n* = 3) before (day zero), during (day one, two) and after (day three, four, six, nine, and 12) drug treatment. (**B**) *MAT1A* expression was quantified via qPCR at each timepoint and expressed in FC. Statistical analysis was conducted using Student’s t-test, *p* < 0.0005 (#). (**C**) Quantitative PCR showing relative *MAT1A* gene expression in *MAT1A* plasmid transfected 5637 bladder cancer cells (5637*^MAT1A^*^+^) expressed as FC, (*n* = 3 biological replicates), *p* < 0.0005 (#) compared to expression at day four in post drug treated 5637 cells (5637^Rx^) (NS, non-significant). (**D**) Representative gel of *MAT1A* protein expression relative to control b-tubulin analyzed via Western blotting using the Jess system (ProteinSimple). (**E**) Normalized *MAT1A*/b-tubulin protein expression calculated using Jess system densitometry expressed as percent of wildtype *MAT1A* expression in 5637 control cells (*n* = 6 biological replicates), *p* < 0.005 (**). (**F**) IC50 gemcitabine drug toxicity curve between empty vector transfected and *MAT1A* transfected cells (*n* = 5 biological replicates per condition per dose).

*MAT1A* expression was next quantified in vitro, in 5637 human bladder cancer cells throughout a 48 h exposure to gemcitabine as well as during a 12 day post treatment recovery period (Figure 4A). Baseline *MAT1A* transcription was low, but gradual increases in *MAT1A* mRNA levels were observed starting at 24 h after the start of treatment with an expression peak two days following the removal of gemcitabine, exclusively in the drug treated (5637^Rx^) samples. After day four, *MAT1A* transcription gradually decreased until day 14, when levels returned to approximately the same baseline expression levels measured prior to drug treatment (Figure 4B).

To further investigate the role of *MAT1A* in tumor relapse following gemcitabine treatment, we overexpressed *MAT1A* in transient transfections of 5637 cells (5637*^MAT1A^*^+^) (Figure 4C) and compared the gemcitabine IC50 profile of these cells to mock transfected controls. 5637*^MAT1A^*^+^ cells had a significant two-fold higher level of *MAT1A* protein than 5637 control cells (Figure 4D,E) and displayed ~100× increases in drug resistance, as evidenced by a change in IC50 concentration from 7.55×10^−6^ mM to 7.95×10^−4^ mM following exposure to a range of gemcitabine drug concentrations (Figure 4F).

### 2.3. MAT1A Confers a Survival Advantage, Potentially Through Decreases in Active Transcription and Proliferation

To obtain mechanistic insights into how *MAT1A* upregulation allows for such increases in drug tolerability as evidenced by dose response analysis (Figure 4F), we conducted RNA-seq on 5637*^MAT1A^*^+^ cells and compared their transcript levels to those from empty vector transfected 5637 control cells (5637) (Figure S2. An ontology analysis was conducted and revealed downregulation in many senescence related pathways (Table S3). Upregulated genes did not show enrichment for any relevant ontology categories.

**Figure 5:**
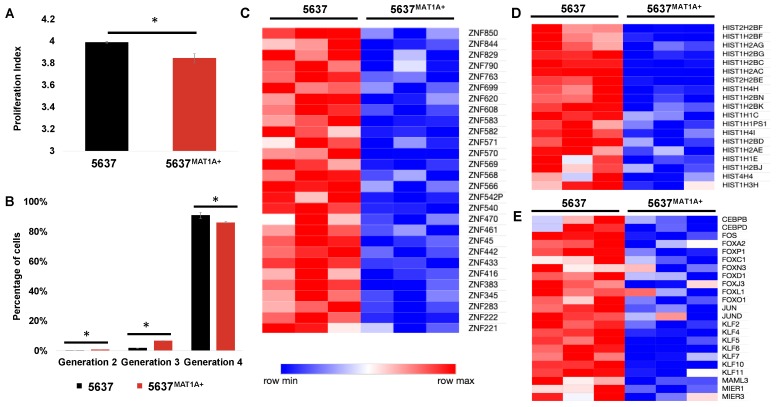
*MAT1A* overexpression decreases cell proliferation and transcriptional activity. (**A**) Proliferation indices over 72 h (* *p* < 0.05) in 5637 and 5637*^MAT1A^*^+^ cells and (**B**) percentage of 5637 versus 5637*^MAT1A^*^+^ cells in various generations following initial cell seeding at generation zero (* *p* < 0.05). Cells were stained with carboxyfluorescein diacetate succinimidyl ester (CFSE) and analyzed using flow cytometry to evaluate changes in proliferation. Data was analyzed and proliferation was modeled in FlowJo^®^. RNA-seq analysis identified whole genome transcriptomic alterations in 5637*^MAT1A^*^+^ and 5637 cells. Heat maps representing differentially expressed genes (*n* = 3 biological replicates) reveal downregulation in three gene groups: (**C**) zinc finger, (**D**) histone, and (**E**) transcription factors.

Gemcitabine is a chemotherapeutic which relies on the highly proliferative nature of cancer cells to exert an anti-cancer effect, thus, we hypothesized that *MAT1A* overexpression could influence proliferative ability. Proliferative indices were calculated via flow cytometric analysis and computational models utilizing FlowJo software. Over four days in culture, *MAT1A* overexpressing cells had a lower proliferation index (PI: 3.84) compared to control (PI: 3.99), (Figure 5A). Percentages of total live cells in each generation of a total cell population were also calculated. In generations two and three, there was a statistically significantly greater percentage of *MAT1A* overexpressing cells compared to control. Additionally, 86% of 5637*^MAT1A^*^+^ cells were found in generation four compared to 91% of control cells (Figure 5B). Further, 70 of 221 (31%) of downregulated genes were transcription factor encoding genes and belonged to protein families associated with a transcriptionally active cell state: Zinc finger motifs, histones, and additional transcription factors (Figure 5C–E).

## 3. Discussion

The cancer field is rapidly moving towards developing new strategies for more effectively prolonging life for those afflicted by highly aggressive cancer types. These include changing the formulation of chemotherapy by prescribing multi-drug regimens instead of one chemotherapeutic at a time. Gemcitabine has been the backbone of neoadjuvant chemotherapy for many cancer subtypes, and it has been undoubtedly an effective strategy for shrinking tumors prior to surgery, contributing to a prolonged life span [13]. However, in a recent clinical trial, patients diagnosed with early stage pancreatic ductal carcinoma treated with FOLFIRINOX, a mixture of four chemotherapy drugs (fluorouracil [5-FU], leucovorin, irinotecan, and oxaliplatin) had a substantially increased survival [14], emphasizing the need to continue to develop multi-drug formulations. Despite these improvements most of these patients continue to succumb to drug relapse [15]. Therefore, understanding the underlying molecular mechanisms of relapse following drug treatment in cancer model systems will provide valuable information to expand the treatment options for patients with re-emerging tumors [16].

Several studies have provided supporting evidence that genes involved in methionine metabolic pathways tend to be altered in a variety of cancer types, hinting that this pathway may be involved in various aspects of tumorigenesis [12,13,14]. Our work strengthens this observation and provides new mechanistic evidence that alterations of this pathway directly affect drug tolerance to gemcitabine and contributes to the emergence of drug relapse in bladder cancer patient derived xenograft models. Here we show that elevated levels of *MAT1A* significantly alter chemosensitivity over a wide range of gemcitabine doses, and that this phenotype was directly mediated by *MAT1A* (Figure 4). Therefore, we may speculate that inhibiting *MAT1A* function in cancer cells might be beneficial in enhancing drug responsiveness. However, future studies will need to address whether attempting to repress *MAT1A* in vivo can improve cancer outcomes [17].

*MAT1A* belongs to a family of methyltransferases that contribute to methylation of a variety of molecules including DNA and histones, via synthesis of SAM [18]. In cancer research, *MAT1A* has been mostly described in the context of hepatocellular carcinoma (HCC), where it was previously shown that a switch in gene expression from *MAT1A* to *MAT2A* (M1-M2 switch) promoted cancer invasion and metastasis. One study showed that enhancing the M1-M2 switch promoted the ability of these cancer cells to metastasize, and this phenomenon was correlated with human data where a balance in favor of M2 (M1 < M2) correlated with increased metastasis and high rate of recurrence in HCC patients. Mechanistically this work highlighted that *MAT1A* was essential for methylating the promoters of certain genes such as osteopontin (OPN), and by repressing *MAT1A* expression such genes were freed from methylation-dependent repression and corroborated with other *MAT1A* activated genes to promote metastasis-driving pathways such as extracellular-signal-regulated kinase (ERK) signaling [19]. Our study however, finds the expression to be tipped in favor of M1 in the bladder, and the downstream outcomes of M1 overexpression may have broader implications including increased survivability in the presence of chemotherapy. Our findings provide novel mechanistic insights into the emergence of drug resistance in bladder cancer. Natively, *MAT1A* has a tissue specific expression pattern with robust expression in liver, pancreas, skin, ovaries, and testis tissues and has been previously characterized in the context of hepatocellular carcinoma [20]. Our findings further highlight that expression of *MAT1A* in extrahepatic tissue associates with poor prognosis. Interestingly, *MAT1A* is not expressed in the urinary bladder under normal conditions, further suggesting the importance of this work as a novel biomarker for bladder cancer (Figure S1). Further research will be needed to evaluate utility of *MAT1A* as a biomarker across other cancer types in tissues where *MAT1A* is not natively expressed, as an indicator of aggressiveness and/or relapse post treatment.

Since *MAT1A* has been previously shown to repress gene expression via DNA methylation of gene promoters, it is possible that the down-regulation of histone genes observed in cells overexpressing *MAT1A* may be caused by direct methylation of these promoters. Alternatively, *MAT1A* may methylate histones and contribute to a regulatory feedback. Since the discovery of RNA methyltransferases a few years ago, this area is now receiving increased attention as a new regulatory mechanism of controlling gene expression [21]. This new layer of regulation is termed “epitranscriptomics.” Similar to *MAT1A*, RNA methyltransferases, demethylases, and m6A-binding proteins are frequently upregulated in human cancer tissues from a variety of organ origins. While their expression levels have not yet been examined in the context of drug resistance, elevated levels of these genes are correlated with cancer progression and metastasis. While the biochemistry of *MAT1A* has been mostly examined in the context of DNA methylation, addition work is required to determine whether this enzyme also contributes to other types of methylation, including RNA and histone protein methylation. Additionally, ultrasensitive quantification of SAM-derived methyl groups will further elucidate differences in DNA, RNA, and histone protein methylation to provide insight into whether SAM-mediated epigenetic modifications through *MAT1A* provide cancer cells with a drug resistant phenotype. DNA methylation, histone modifications, and the chromatin structure are profoundly altered in human cancers and future studies will further determine how *MAT1A* is involved in some of these processes to contribute to drug resistance, in bladder, and possible other types of cancer. The work presented here has clinical relevance as well as applications in basic research. Evidence of *MAT1A* in bladder tumors may prove as a powerful prognostic tool to evaluate surgical margins and indicate likelihood of relapse in histopathology samples. Lastly, association of *MAT1A* with drug resistant cell populations may also lead to new small molecule inhibitors capable of eliminating drug resistant cells that ultimately allow tumor relapse. 

## 4. Materials and Methods

### 4.1. Bladder Cancer Patient Derived Xenografts

Bladder cancer xenografts (BL0293 and BL0440) were previously described with corresponding drug sensitivity data [9]. Frozen samples from Passage 4 were propagated in NSG (NOD.Cg-Prkdcscid Il2rgtm1Wjl/SzJ) immunodeficient mice (The Jackson Laboratory, Bar Harbor, ME, USA) by injecting single cell suspensions (3 × 10^6^ tumor cells administered in 100 µL volume of cell suspension in 1:1 Matrigel (Corning)) subcutaneously (SQ) into the right flank. Tumor burden was assessed by measuring tumor length and width using digital calipers twice per week for up to 10 weeks. Tumor volume was estimated using the formula (length*width^2^)/2. Tumors were excised when they reached an estimated volume of 1 cm^3^ or when other IACUC criteria were met. All animal experiments were approved by the Lawrence Livermore National Laboratory Institutional Animal Care and Use Committee and conform to the Guide for the care and use of laboratory animals. Protocol 168 was approved October 14, 2015 by LLNL IACUC to conduct this work.

### 4.2. Generation of Drug Relapsed PDX Tumors

Gemcitabine hydrochloride (BioVision, Inc, Milpitas, CA, USA) was dissolved in PBS at a final concentration of 25 mg/mL and administered intraperitoneally (IP) once per week for four consecutive weeks at 150 mg/kg. Concurrently, cisplatin (Spectrum Chemical Mfg Corp, New Brunswick, NJ, USA) was resuspended in PBS at 1 mg/mL and delivered intravenously (IV) at 2 mg/kg per animal. Cisplatin dosing occurred on days one, two, and three and days 15, 16, and 17 of drug treatment. Control animals (*n* = 3) were treated with an equivalent amount of PBS at the same time as chemotherapy treatment. Tumors were grown in vivo for up to 65 days (*n* = 5).

### 4.3. RNA Sequencing

Harvested tumors were minced and manually dissociated using a 40 µm cell strainer. Tumor cells were flash frozen in liquid nitrogen and stored at −80 °C until processed. RNA was isolated from 6 x 10^6^ homogenized cells by using QIAshredder and RNeasy^®^ Mini Qiagen kits (Hilden, Germany). RNA concentrations were quantified using a Qubit^®^ RNA HS Assay Kit (ThermoFisher Scientific, Waltham, MA, USA). Quality was assessed before sequencing using an RNA 6000 Nano kit run on an Agilent 2100 Bioanalyzer (Agilent Technologies Inc., Santa Clara, CA, USA). cDNA libraries were prepared from 100 ng of total isolated RNA per manufacturer specification from BL0293/BL0440 in vivo tumors or in vitro 5637 bladder cancer cells for RNA sequencing using Illumina TruSeq RNA Library Preparation kit version 2.0 (Illumina Inc., San Diego, CA, USA) and run on an Illumina Nextseq 500 using the High Output 75 cycles kit (Illumina Inc., San Diego, CA, USA).

### 4.4. RNA Sequencing Data Analysis

Read quality from RNA-seq raw data was first assessed using FastQC [22]. Sequence data was aligned to the human genome (hg19) using TopHat2 and STAR [23]. Subsequently, gene-wise read counts were calculated using ‘featureCounts’ from Rsubread package. Then, the count data was normalized using TMM normalization method, and differentially expressed genes were identified using ‘limma’ [24] and ‘voom’ [25]. Genes that were >1.5 fold up- or down-regulated with an FDR corrected *p*-value <0.05 were considered significantly differentially expressed. Pathway ontology analysis was conducted using ToppGene tool using *p*-value < 0.05 to denote process significance [26].

### 4.5. Plasmid Transfection Generating 5637^MAT1A+^ Cells

5637 bladder cancer cell line was obtained from ATCC. *MAT1A* plasmid (Origene; NM_000429 Human Untagged Clone, Product #SC119881) was isolated and purified using MidiPrep™ kit (Qiagen Inc., Hilden, Germany). Subconfluent 5637 cells were transfected with *MAT1A* plasmid DNA (2 µg/µL) using a Lipofectamine 3000 kit following manufacturer guidelines (ThermoFisher Scientific, Waltham, MA, USA). Briefly, liposomes containing plasmid DNA were constructed in low serum media and added to cells of interest. Transfection occurred over 48 h, followed by downstream experimental procedure. Control experiments were carried out with an empty expression vector.

### 4.6. Immunohistochemical Analysis

PDX tumors were prepared for IHC analysis by fixation in 10% neutral buffered formalin for three to four days. Tumors were stored in 70% isopropanol until ready for embedding in Type IV paraffin. Tumors were sectioned using a Leica Biosystem RM2125 microtome to collect 0.2 µm tissue sections. Sections were counterstained with hematoxylin and protein of interest was probed using *MAT1A* primary antibody (ab174687) and labeled streptavidin biotin (LSAB) secondary antibody.

### 4.7. Human Patient Tissue Microarray Analysis (TMA)

Human bladder cancer tissue microarray (TMA) sections were obtained from the Biorepository at UC Davis Comprehensive Cancer Center. Patient characteristics are described in Table S3. TMAs were stained for *MAT1A* expression using previously described methods in ‘Immunohistochemical Analysis’ section. Each patient tissue was used in triplicate in the TMA, and were scored individually in the nucleus and the cytoplasm, as 0, 0.25, 0.5, 0.75, 1.0, or 1.5, representing increasing fraction of cells staining for *MAT1A*.

### 4.8. The Human Protein Atlas: MAT1A

*MAT1A* expression in normal human liver and bladder tissues was used for comparison to expression in bladder tumors, shown in this study (Figure S1). *MAT1A* expression in bladder and liver tissue made available through v19.proteinatlas.org, available from http://www.proteinatlas.org [11]. Figure S1B available through https://www.proteinatlas.org/ENSG00000151224-MAT1A/tissue/liver#img. Figure S1C available through: https://www.proteinatlas.org/ENSG00000151224-MAT1A/tissue/urinary+bladder#img.

### 4.9. Western Blotting and Protein Analysis

Proteins were isolated from flash frozen tumor cells or 5637 cells in RIPA lysis buffer (Sigma Aldrich Corporation, St. Louis, MO, USA) followed by centrifugation at 14,000 rcf for 5 min. The supernatants were collected and analyzed using the Jess automated Western blotting system (ProteinSimple, San Jose, CA, USA). Jess reagents (biotinylated molecular weight marker, streptavidin-HRP fluorescent standards, sample buffer, DTT, stacking matrix, separation matrix, running buffer, wash buffer, matrix removal buffer, fluorescent labeled secondary antibodies, antibody diluent, and capillaries) were used according to the manufacturer’s standard protocol. Antibodies were diluted with ProteinSimple antibody diluent as follows: *MAT1A* primary antibody (undiluted, Abcam, Cambridge, United Kingdom, ab174687), beta-Tubulin (1:100, LI-COR Biosciences, Lincoln, ME, Catalog no. 926-42211). Protein concentration was quantified using Compass for SW 4.0 software. Target protein abundance is normalized to the expression of beta-tubulin.

### 4.10. In Vitro Toxicity

5637*^MAT1A^*^+^ and 5637 cells were dosed with a range of gemcitabine concentrations from 0 mM to 30 mM for 48 h post plasmid transfection. Cell viability was assessed at 48 h post drug treatment using an ATP-based cell viability assay following manufacturer guidelines (CellTiterGlo 2.0, Promega Corporation, Madison, WI, USA). IC50 values were calculated using Prism 8 (GraphPad Software Inc., La Jolla, CA, USA).

### 4.11. Quantitative PCR (qPCR)

Total RNA was isolated using a commercially available RNeasy Mini Kit (Qiagen, Hilden, Germany). RNA was reverse transcribed to generate cDNA using a SuperScript IV First Strand Synthesis Kit (ThermoFisher Scientific). Cycling was performed using a Fast Real-Time System on an Applied Biosystems 7900HT instrument. Cycling conditions using SYBR as a DNA dye are as follows: 50 °C for 2 min, 95 °C for 2 min followed by 40 cycles of 95 °C for 3 s then 60 °C for 40 s. Data was normalized to Ct values of *GAPDH* (control gene) and reported as fold change, calculated using the standard ddCt method. *GAPDH* primer sequences: Forward, GTCTCCTCTGACTTCAACAGCG; reverse, ACCACCCTGTTGCTGTAGCCAA. *MAT1A* primer sequences: Forward, TCATGTTCACATCGGAGTCTGT; reverse, CATGCCGGTCTTGCACACT.

### 4.12. Cell Proliferation

5637 cells transfected with *MAT1A* plasmid or a control empty vector were seeded at 20% confluency (*n* = 6) and dyed using the CellTrace™ CFSE Cell Proliferation Kit, to quantify cell proliferation. All cells were collected three days post-transfection and immediately analyzed for fluorescence using a FACSMelody™ flow cytometer (Becton, Dickinson & Co, Franklin Lakes, NJ). Expansion indices as well as proliferation/division indices were determined using cell proliferation modeling generated by FlowJo flow cytometry software (FlowJo, LLC, Ashland, OR, USA). Statistics were performed using a Student’s *t*-test including *n* = 6 biological replicates from each 5637 and 5637*^MAT1A^*^+^ cells.

### 4.13. Statistical Analysis

In vitro analysis: Data is presented as averages +/− standard deviation. Student’s *t*-test was used to calculate statistical significance in Microsoft Excel. TMA analysis of human tissues: Median of three values from each patient were first calculated and then mean and standard deviation derived in each group. *MAT1A* expression was compared between patients with and without prior treatment or statin or between those with chemotherapy or BCG using Mann-Whitney non-parametric tests. Analyses were conducted using GraphPad Prism 8.2. *p* values of less than 0.05 were deemed statistically significant.

## Figures and Tables

**Table 1:** Top 20 upregulated genes in drug relapsed PDX tumors.

Ranking	Gene Symbol	Gene Name	BL0293_FC	BL0440_FC
1	*MAGEA11*	Melanoma-Associated Antigen 11	2.75	8.50
2	*MAT1A*	Methionine Adenosyltransferase 1A	6.66	3.40
3	*DIO2*	Iodothyronine Deiodinase 2	3.04	6.72
4	*PWWP2B*	PWWP Domain-Containing Protein 2B	4.91	3.67
5	*PAGE2*	Prostate-Associated Gene 2 Protein	4.68	2.67
6	*FAM133A*	Family With Sequence Similarity 133 Member A	4.25	2.81
7	*ABHD17C*	Abhydrolase Domain Containing 17C	2.37	4.53
8	*LINC00839*	Long Intergenic Non-Protein Coding RNA 839	2.75	3.62
9	*OAS1*	2′-5′-Oligoadenylate Synthetase 1	1.63	4.56
10	*RSAD2*	Radical S-Adenosyl Methionine Domain Containing 2	2.49	3.28
11	*CMPK2*	Cytidine/Uridine Monophosphate Kinase 2	1.80	3.67
12	*HMGN5*	High Mobility Group Nucleosome Binding Domain 5	2.80	2.49
13	*ZNF300P1*	Zinc Finger Protein 300 Pseudogene 1	2.64	1.91
14	*SEPT14*	Septin 14	1.75	2.57
15	*CEBPA*	CCAAT Enhancer Binding Protein Alpha	2.09	1.81
16	*KCNC4*	Potassium Voltage-Gated Channel Subfamily C Member 4	1.66	2.19
17	*NNMT*	Nicotinamide *N*-Methyltransferase	1.52	2.23
18	*MPC1*	Mitochondrial Pyruvate Carrier 1	1.69	1.71
19	*ZNF385B*	Zinc Finger Protein 385B	1.70	1.52
20	*CEBPD*	CCAAT Enhancer Binding Protein Delta	1.57	1.60

**Table 2:** Pathway ontologies of upregulated genes in relapsed, drug treated BL0293 and BL0440 PDX tumors using ToppGene ToppFun suite.

Reactome Identifier	Name	*p*-Value
R-HSA-909733	Interferon alpha/beta signaling	1.324 × 10^-5^
R-HSA-2408508	Metabolism of ingested SeMet, Sec, MeSec into H2Se	2.084 × 10^-5^
R-HSA-381340	Transcriptional regulation of white adipocyte differentiation	2.142 × 10^-5^
R-HSA-156581	Methylation	6.927 × 10^-5^
R-HSA-71291	Metabolism of amino acids and derivatives	2.613 × 10^-3^
R-HSA-156580	Phase II conjugation	3.383 × 10^-3^
R-HSA-2408522	Selenoamino acid metabolism	4.137 × 10^-3^

## Supplementary Materials

Supplementary materials can be found at https://www.mdpi.com/1422-0067/20/20/4983/s1.

## Abbreviations

MAT1A	Methionine adenosyltransferase 1a
PDX	Patient-derived xenograft
SAM	S-adenosylmethionine
CIS	cisplatin
GEM	gemcitabine
NSG	NOD *scid* gamma
Rx	drug treated
PBS	phosphate buffered saline
FC	fold change
ATP	adenosine triphosphate
PCR	polymerase chain reaction
CFSE	carboxyfluorescein diacetate succinimidyl ester
HCC	Hepatocellular carcinoma
OPN	osteopontin
SQ	subcutaneous
IHC	immunohistochemical
TMA	tissue microarrays
FOLFIRINOX	Leucovorin, fluorouracil, irinotecan, oxaliplatin
BCG	Bacillus Calmette-Guerin therapy
