# Supplementary material for: Methionine Adenosyltransferase 1a (MAT1A) Enhances Cell Survival During Chemotherapy Treatment and is Associated with Drug Resistance in Bladder Cancer PDX Mice
Source: Int J Mol Sci. 2019 Oct 9;20(20):4983. doi: 10.3390/ijms20204983 (PMC6829260; doi:10.3390/ijms20204983)
Supplement: Supplementary file 1 [file ijms-20-04983-s001.pdf]

International Journal of
Molecular Sciences
MDPI

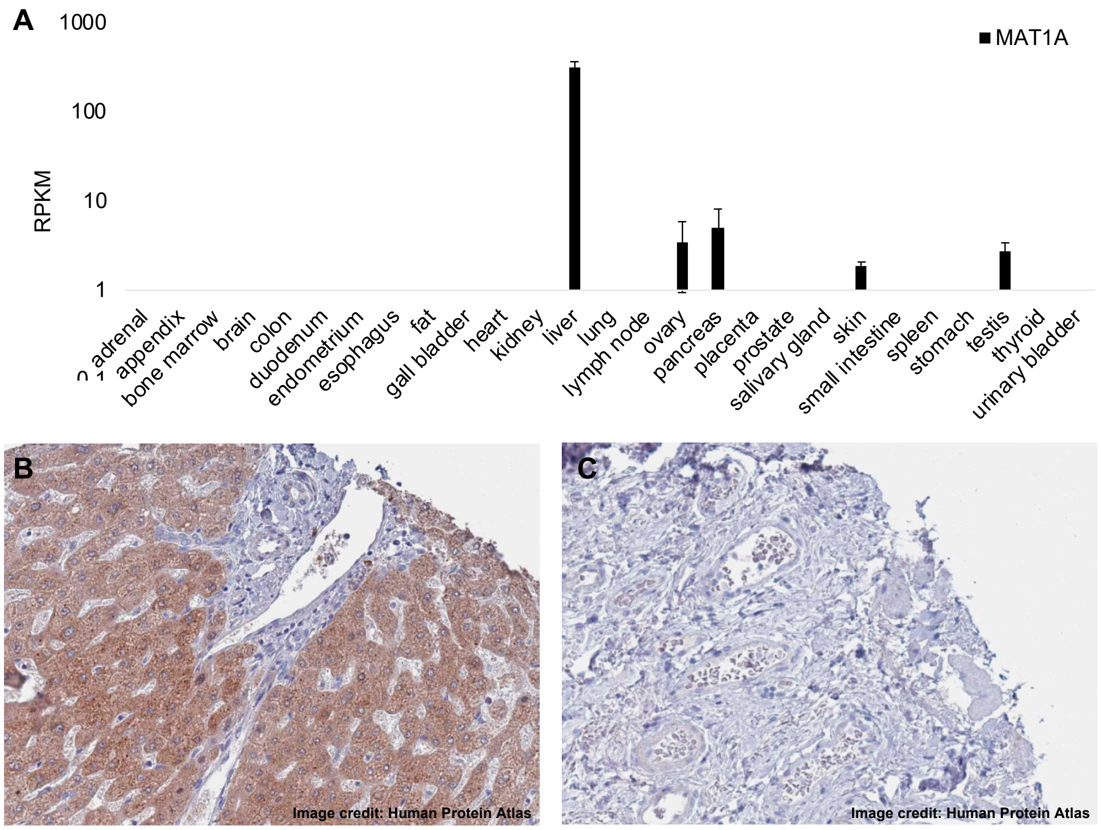

Figure S1. Distribution of methionine adenosyltransferase 1a (***MAT1A*****) expression in healthy human tissues and in bladder cancer.** (A) RNA sequencing (RNA-seq) profiling *MAT1A* expression in normal human tissues, values obtained from Fagerberg et al. [10]. (B) Immunohistochemistry (IHC) of normal liver tissue obtained from The Human Protein Atlas, available from http://www.proteinatlas.org, positive for *MAT1A* expression [11]. (C) IHC of normal bladder tissue obtained from The Human Protein Atlas, available from http://www.proteinatlas.org, negative for *MAT1A* expression [11].

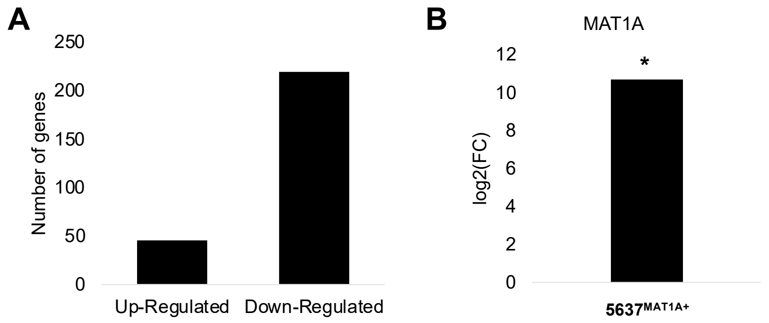

Figure S2. **RNA-seq profiling of 5637^{MAT1A+} cells.** (A) Total number of up- and down-regulated genes in 5637 cells overexpressing *MAT1A* (5637^{MAT1A+}) compared to empty vector transfected control cells (5637), n=3 biological replicates. (B) *MAT1A* gene expression expressed in $\log_2$ fold change based on RNA sequencing data, FDR<0.05(*).

Table S1. Top 20 down-regulated genes in drug relapsed patient-derived bladder cancer xenograft (PDX) tumors.

Ranking	Gene Symbol	Gene Name	BL0293_FC	BL0440_FC
1	*SNORD14C*	Small Nucleolar RNA, C/D Box 14C	-3.26	-1.61
2	*SNORD31*	Small Nucleolar RNA, C/D Box 31	-2.53	-1.91
3	*KRT15*	Keratin 15	-2.43	-1.85
4	*JMJD7-PLA2G4B*	Jumonji Domain Containing 7-Phospholipase A2, Group IVB (Cytosolic) Read-Through	-2.64	-1.63
5	*PITRM1-AS1*	PITRM1 Antisense RNA 1	-2.46	-1.61
6	*MLXIPL*	MLX Interacting Protein Like	-2.10	-1.95
7	*ANXA5*	Annexin A5	-2.07	-1.80
8	*SNORD29*	Small Nucleolar RNA, C/D Box 29	-1.81	-2.04
9	*LPIN3*	Lipin 3	-2.16	-1.67
10	*PDXDC2P*	Pyridoxal Dependent Decarboxylase Domain Containing 2, Pseudogene	-2.21	-1.60
11	*PRRT2*	Proline Rich Transmembrane Protein 2	-1.86	-1.74
12	*DDX12P*	DEAD/H-Box Helicase 12, Pseudogene	-2.02	-1.53
13	*GNRH1*	Gonadotropin Releasing Hormone 1	-2.03	-1.51
14	*C10orf55*	Chromosome 10 Open Reading Frame 55	-1.82	-1.55
15	*C11orf68*	Chromosome 11 Open Reading Frame 68	-1.64	-1.73
16	*NLRP1*	NLR Family Pyrin Domain Containing 1	-1.74	-1.60
17	*P2RX7*	Purinergic Receptor P2X 7	-1.54	-1.51

Table S2. **Patient characteristics.** 55 patients' tissues were used in this study and their characteristics are described below. The patients were all diagnosed with bladder cancer and underwent radical cystectomy for the treatment of bladder cancer.

	N	55	
**Race**	Caucasian	35	(63.64%)
	Asian	1	(1.82%)
	African American	1	(1.82%)
	Unknown	18	(32.73%)
**Ethnicity**	Hispanic/Latino	4	(7.27%)
	Not Hispanic/Latino	34	(61.82%)
	Unknown	17	(30.91%)
**Age**	Median	68	(36-85)
**Gender**	Male	45	(81.82%)
	Female	10	(18.18%)
**Type**	Transitional Cell Carcinoma	28	(50.91%)
	Urothelial Carcinoma	23	(41.82%)
	Papillary/Squamous Cell Carcinoma	4	(7.27%)
**Grade**	High Grade	23	(41.82%)
	Intermediate Grade	23	(41.82%)
	Low Grade	4	(7.27%)
	not reported/undetermined	5	(9.09%)
**Neoadjuvant Treatment**	BCG	4	(7.27%)
	Chemotherapy	11	(20.00%)
	None Reported	40	(72.73%)

Table S3. Top pathway ontologies downregulated in 5637^{MAT1A+} overexpressing cells identified using ToppGene.

Name	pValue
DNA Damage/Telomere Stress Induced Senescence	3.971e-17
Cellular Senescence	1.262e-15
Condensation of Prophase Chromosomes	8.061e-15

Senescence-Associated Secretory Phenotype (SASP)	1.307e-14
HDACs deactylate histones	1.451e-14
RNA Polymerase I Promoter Opening	1.735e-14
Packaging of Telomere Ends	2.368e-14
DNA methylation	2.750e-14
Activated PKN1 stimulates transcription of AR (androgen receptor) regulated genes KLK2 and KLK3	4.290e-14
SIRT1 negatively regulates rRNA Expression	6.592e-14
